# Predicting the occurrence of embolic events: an analysis of 1456 episodes of infective endocarditis from the Italian Study on Endocarditis (SEI)

**DOI:** 10.1186/1471-2334-14-230

**Published:** 2014-04-29

**Authors:** Marco Rizzi, Veronica Ravasio, Alessandra Carobbio, Irene Mattucci, Massimo Crapis, Roberto Stellini, Maria Bruna Pasticci, Pierangelo Chinello, Marco Falcone, Paolo Grossi, Francesco Barbaro, Angelo Pan, Pierluigi Viale, Emanuele Durante-Mangoni

**Affiliations:** 1 USC Malattie Infettive, Ospedale Papa Giovanni XXIII, piazza OMS 1, Bergamo, BG 24127, Italia; 2 Fondazione per la Ricerca Ospedale Maggiore (FROM), Ospedale Papa Giovanni XXIII, Bergamo, Italia; 3 Cattedra di Medicina Interna ed UOC Medicina Infettivologica e dei Trapianti, Seconda Università di Napoli, AO Monaldi, Napoli, Italia; 4 Clinica di Malattie Infettive, AOU Santa Maria della Misericordia, Udine, Italia; 5 Clinica di Malattie Infettive e Tropicali, Università degli Studi di Brescia, Brescia, Italia; 6 Sezione Clinica delle Malattie Infettive, Dipartimento Medicina Sperimentale e Scienze Biochimiche, Università di Perugia, Perugia, Italia; 7 Istituto Nazionale per le Malattie Infettive Spallanzani, Roma, Italia; 8 Dipartimento di Salute Pubblica e Malattie Infettive – Dipartimento di Emergenza, Policlinico Umberto I, Università degli Studi di Roma “La Sapienza”, Roma, Italia; 9 Clinica di Malattie Infettive e Tropicali, Università degli Studi dell’Insubria, Varese, Italia; 10 UO Malattie Infettive e Tropicali, Azienda Ospedaliera di Padova, Padova, Italia; 11 UO Malattie Infettive, Istituti Ospitalieri di Cremona, Cremona, Italia; 12 Unità Operativa di Malattie Infettive, Policlinico S. Orsola-Malpighi, Alma Mater Studiorum Università di Bologna, Bologna, Italia

**Keywords:** Infective endocarditis, Embolism, Stroke, Risk score

## Abstract

**Background:**

Embolic events are a major cause of morbidity and mortality in patients with infective endocarditis. We analyzed the database of the prospective cohort study SEI in order to identify factors associated with the occurrence of embolic events and to develop a scoring system for the assessment of the risk of embolism.

**Methods:**

We retrospectively analyzed 1456 episodes of infective endocarditis from the multicenter study SEI. Predictors of embolism were identified. Risk factors identified at multivariate analysis as predictive of embolism in left-sided endocarditis, were used for the development of a risk score: 1 point was assigned to each risk factor (total risk score range: minimum 0 points; maximum 2 points). Three categories were defined by the score: low (0 points), intermediate (1 point), or high risk (2 points); the probability of embolic events per risk category was calculated for each day on treatment (day 0 through day 30).

**Results:**

There were 499 episodes of infective endocarditis (34%) that were complicated by ≥ 1 embolic event. Most embolic events occurred early in the clinical course (first week of therapy: 15.5 episodes per 1000 patient days; second week: 3.7 episodes per 1000 patient days). In the total cohort, the factors associated with the occurrence of embolism at multivariate analysis were prosthetic valve localization (odds ratio, 1.84), right-sided endocarditis (odds ratio, 3.93), *Staphylococcus aureus* etiology (odds ratio, 2.23) and vegetation size ≥ 13 mm (odds ratio, 1.86). In left-sided endocarditis, *Staphylococcus aureus* etiology (odds ratio, 2.1) and vegetation size ≥ 13 mm (odds ratio, 2.1) were independently associated with embolic events; the 30-day cumulative incidence of embolism varied with risk score category (low risk, 12%; intermediate risk, 25%; high risk, 38%; p < 0.001).

**Conclusions:**

*Staphylococcus aureus* etiology and vegetation size are associated with an increased risk of embolism. In left-sided endocarditis, a simple scoring system, which combines etiology and vegetation size with time on antimicrobials, might contribute to a better assessment of the risk of embolism, and to a more individualized analysis of indications and contraindications for early surgery.

## Background

Embolic events are common complications of infective endocarditis (IE) and are of prognostic importance [1-3]. Neurologic complications, mostly caused by embolic events, are frequent causes of intensive care unit admission, and embolism is a strong predictor of death for patients who have IE [4-8].

Small, clinically inapparent emboli probably occur in most patients who have IE, as demonstrated with positron-emission tomography and magnetic resonance imaging; silent cerebrovascular complications (including ischemia and microhemorrhage) may occur in up to 81% of patients [9-11]. Symptomatic embolism may occur in 13% to 46% of patients [6,12,13]. Many symptomatic emboli involve the central nervous system; clinical evidence of cerebrovascular embolic events is present in 12% to 40% of patients who have IE; the incidence may be higher in patients who are treated in referral centers, critically ill, or admitted to an intensive care unit [5,6,14].

Assessment of the embolic risk in individual patients is difficult, this having a major impact on clinical decisions regarding the indications for diagnostic procedures, antiaggregant and anticoagulant therapy and the timing of cardiac valve surgery [2,3,12,15-20]. Prevention of embolism is an established indication for surgery; in the Euro Heart Survey, vegetation size and previous embolism were reported as factors contributing to the surgical decision in, respectively, 48% and 18% of the patients who had surgery; in the French series recently published by Hubert et al. the presence of large vegetations (with or without previous embolism) was reported as an indication for surgery in 36% of the 493 patients who underwent surgery [3,21,22].

Most previous studies about the embolic complications of IE have been retrospective, with selection bias and limited numbers of patients. In addition, the epidemiology and standard of care of IE have clearly evolved over the years, and less recent observations may no more adequately represent the contemporary clinical profile of the disease [8,12,23-26]. The present study was based on data from a large prospective observational cohort; the retrospective analysis of the database had two main objectives: (1) to identify factors associated with the occurrence of embolic events and (2) to design a scoring system for the assessment of the risk of embolic complications.

## Methods

### Patients

The Italian Study on Endocarditis (Studio Endocarditi Italiano - SEI) working group was established in October 2003 in order to promote research on IE in Italy. Within this group, a prospective, observational, multicenter, open cohort study was designed. All consecutive episodes of IE diagnosed from January 2004 through December 2011 at 25 secondary and tertiary care institutions were included in the study. The diagnosis of IE was defined as possible or definite, in accordance with the modified Duke criteria [27]. Adult and pediatric patients were identified prospectively using institution-specific procedures, so as to ensure the consecutive enrollment of all the patients with IE observed at each participating institution. Echocardiography, diagnosis of embolic complications and treatment, were in accordance with current clinical practice (the study did not prescribe a standard diagnostic and therapeutic approach).

At the time of clinical observation of each patient, clinical data were recorded in a form that was designed for the study and registered in an electronic data entry system. The SEI database was maintained at the Infectious Diseases Department, Bergamo Hospital; the study was approved by the ethics committee “Comitato di Bioetica dell'Azienda Ospedali Riuniti di Bergamo”, and patients consented to enrollment in the SEI cohort as required by local guidelines and procedures. Details on the SEI study have been previously published [28].

Here we present the results of a retrospective analysis of the SEI database, aimed at investigating factors associated with the occurrence of embolic events; all the episodes of possible and definite IE were included.

### Clinical data

Available information included demographic data, predisposing conditions, anatomic site of disease, microbiology, echocardiographic findings (presence, number, location and maximal length of vegetations), antimicrobial and surgical treatment, complications (heart failure, embolic events, intracardiac abscess, persistent positive blood cultures and new cardiac conduction abnormalities) and clinical outcome (survival and New York Hearth Association functional class). Central nervous system embolic events were reported as transient ischemic attack, ischemic stroke or hemorrhagic stroke. Peripheral embolic events were reported with details of anatomic site. Mycotic aneurysms, meningitis, encephalopathy, cerebral abscesses, arthritis, spondylodiscitis and cutaneous complications were excluded.

Infective endocarditis was classified as left-sided IE (LS-IE), left-sided native valve IE (LS-NVE), left-sided prosthetic valve IE (LS-PVE), right-sided IE (RS-IE) or IE associated with a cardiac implantable electronic device (CIED-IE).

### Statistical analysis

Data analysis was performed with statistical software (Stata 13, StataCorp LP, College Station, TX). Comparisons were done using χ^2^ test (chi-square test) for proportions and Mann–Whitney test for ordered or continuous variables. Receiver operating characteristic (ROC) analysis was performed to determine the optimal vegetation size cut-off for the prediction of embolism.

Cumulative incidence analysis was performed from the date of starting treatment to the date of embolism (uncensored) or discharge from the hospital (censored). Cardiac surgery was treated as a competing event. Univariate and multivariate analyses were performed with logistic regression. Variables that were statistically significant in univariate analysis, variables that were clinically meaningful and possible confounders, were selected for prognostic assessment. For LS-IE, a weight was assigned to each factor that was associated with embolism, in order to build a risk score. The Kaplan‒Meier method was used to estimate univariate cumulative incidence of embolism according to the risk score. All tests were 2-sided, and statistical significance was defined by p ≤ 0.05. The probability of the occurrence of embolic events for each risk category was calculated for each day on treatment (from day 1 through day 30).

## Results

### Clinical characteristics of patients and embolic events

During the study period, 1456 IE episodes were observed at the participating institutions (1297 definite and 159 possible episodes); 10 episodes (0.7%) involved pediatric patients (age <18 y); basic demographic and medical data are summarized in Table 1.

**Table 1:** Demographic and clinical characteristics of the cohort*

**Characteristics**	**All episodes**	**LS-NVE**	**LS-PVE**	**RS-IE**	**CIED-IE**
Number of IE episodes	1456 (100)	967 (66.4)	339 (23.3)	89 (6.1)	61 (4.2)
Age (y)	65 (50 to 64)	64 (48 to 74)	70 (61 to 76)	48 (35 to 67)	67 (55 to 77)
Male	1018 (70)	688 (71)	221 (65)	61 (69)	48 (79)
Comorbidity and predisposing conditions					
Native valve disease	372 (26)	299 (31)	60 (18)	7 (8)	6 (10)
Diabetes mellitus	268 (18)	164 (17)	79 (23)	10 (11)	15 (25)
Chronic liver disease	262 (18)	169 (18)	49 (15)	38 (43)	6 (10)
Cancer	175 (12)	131 (14)	28 (8)	10 (11)	6 (10)
Chronic intravenous access	154 (11)	102 (11)	34 (10)	11 (12)	7 (12)
Current intravenous drug abuse	131 (9)	81 (8)	14 (4)	34 (38)	2 (3)
Previous IE	118 (8)	48 (5)	59 (17)	7 (8)	4 (7)
Congenital heart disease	112 (8)	85 (9)	16 (5)	6 (7)	5 (8)
Immunosuppressive therapy	86 (6)	70 (7)	11 (3)	2 (2)	3 (5)
HIV infection	52 (4)	36 (4)	4 (1)	10 (11)	2 (3)
Hemodialysis	29 (2)	21 (2)	4 (1)	2 (2)	2 (3)
Microbiology					
*Staphylococcus aureus*	283 (19)	181 (19)	53 (16)	35 (39)	14 (23)
Viridans group streptococci	217 (15)	172 (18)	29 (9)	8 (9)	8 (13)
*Enterococcus* species	182 (13)	117 (12)	53 (16)	8 (9)	4 (7)
Coagulase-negative staphylococci	147 (10)	74 (8)	55 (16)	4 (5)	14 (23)
*Streptococcus bovis*	142 (10)	105 (11)	29 (9)	7 (8)	1 (2)
Other streptococci	59 (4)	46 (5)	7 (2)	3 (3)	3 (5)
Other microorganisms	61 (4)	36 (4)	20 (6)	2 (2)	3 (5)
Polymicrobial	51 (4)	34 (4)	10 (3)	4 (5)	3 (5)
Fungi	20 (1)	12 (1)	8 (2)	0 (0)	0 (0)
HACEK	3 (0.2)	0 (0)	2 (0.6)	1 (1)	0 (0)
Microbiology negative	291 (20)	190 (20)	73 (22)	17 (19)	11 (18)

*Data reported as number, median (interquartile range), or number (% patients).

*Abbreviations:CIED-IE* infective endocarditis associated with a cardiac implantable electronic device, *HACEKHaemophilus species*, *Actinobacillus actinomycetemcomitans*, *Cardiobacterium hominis*, *Eikenella corrodens*, and *Kingella kingae*, *HIV* human immunodeficiency virus, *LS-NVE* left-sided native valve endocarditis, *LS-PVE* left-sided prosthetic valve endocarditis, *RS-IE* right-sided infective endocarditis.

**Figure 1:**
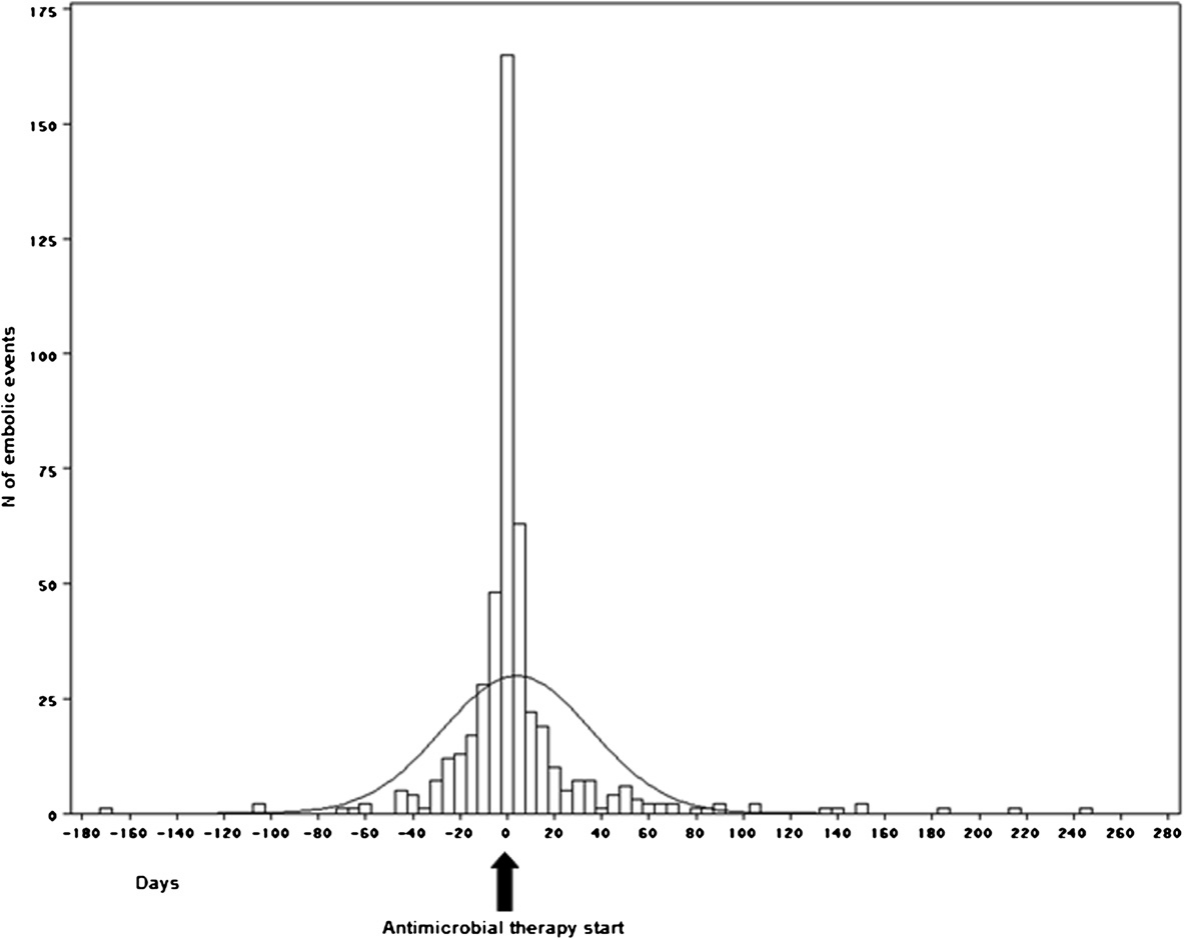
**Timing of embolic events.** Negative values represent days before the beginning of the antibiotic therapy; zero represents the day of the beginning of antibiotic therapy; positive values represent the days after the beginning of the antibiotic therapy.

Embolization occurred in 499 IE episodes (34.3%), with more than one embolic event in 135 episodes (9.3%), and a cumulative number of 700 embolic events (Table 2). In 121/499 IE episodes (24.2%) the first embolic event occurred in the days preceding the diagnosis of IE and the start of antimicrobial therapy; in 77 cases (15.4%) on the same day of diagnosis. After the starting of antimicrobial therapy, most embolic events occurred early (15.5 episodes per 1000 patient days during the first week of therapy; 3.7 episodes per 1000 patient days during the second week of therapy) (Figure 1). In 93 IE episodes, the disease was complicated by both CNS and peripheral embolism; in 25 of these episodes CNS embolism was preceded by overt peripheral embolism.

**Table 2:** Type and anatomic site of embolic events*

**Characteristics**	**All IE episodes**	**LS-NVE**	**LS-PVE**	**RS-IE**	**CIED-IE**
Number of IE episodes	1456	967	339	89	61
Embolic events	700	436	166	74	24
CNS emboli	249 (36)	174 (40)	68 (41)	3 (4)	4 (17)
Ischemic stroke	176 (25)	126 (29)	45 (27)	2 (3)	3 (13)
Transient ischemic attack	42 (6)	26 (6)	14 (8)	1 (1)	1 (4)
Hemorrhagic stroke	31 (4)	22 (5)	9 (6)	0 (0)	0 (0)
Peripheral emboli	451 (64)	262 (60)	98 (59)	71 (96)	20 (83)
Pulmonary	122 (17)	26 (6)	25 (15)	57 (84)	14 (58)
Splenic	117 (17)	90 (21)	23 (14)	3 (4)	1 (4)
Limbs	95 (14)	70 (16)	15 (9)	7 (10)	3 (13)
Other	117 (17)	76 (17)	35 (21)	4 (6)	2 (8)

*Data reported as number or number (% of embolic events).

*Abbreviations*: *CIED-IE* infective endocarditis associated with a cardiac implantable electronic device, *LS-NVE* left-sided native valve endocarditis, *LS-PVE* left-sided prosthetic valve endocarditis, *RS-IE* right-sided infective endocarditis.

IE episodes complicated by embolic events had a higher in-hospital mortality (OR 1.84; 95% CI 1.36 – 2.49, p ≤ 0.0001); no significant difference was observed in the rate of cardiac surgical procedures when comparing IE episodes complicated by embolism with uncomplicated IE episodes (OR 0.98; 95% CI 0.79 – 1.23, p ns) (Additional file 1: Appendix 1 and Additional file 2: Appendix 2).

### Factors associated with embolic events

**Figure 2:**
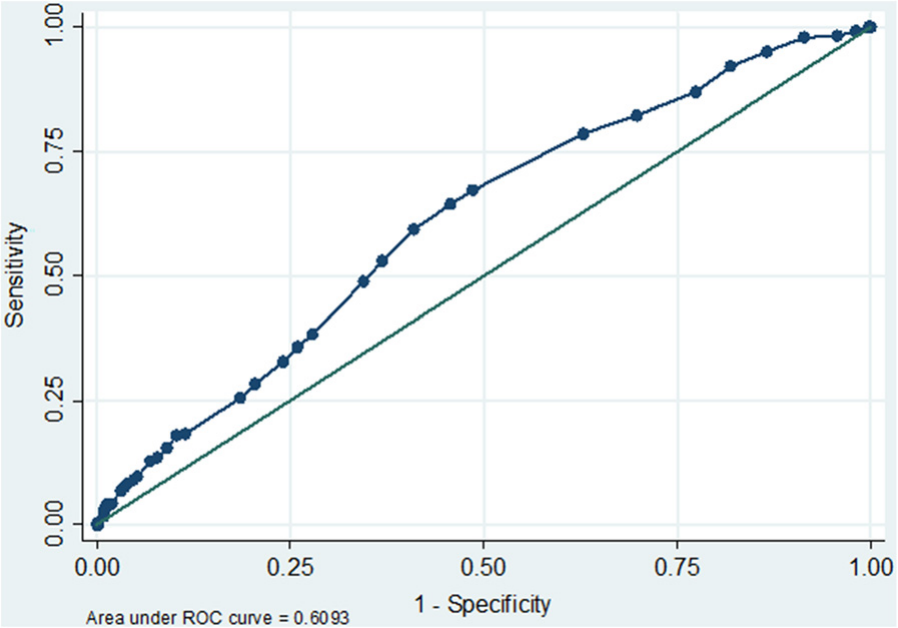
**Total cohort: size of vegetation.** Receiver operating characteristic analysis for the prediction of embolic events, based on vegetation size.

In the total cohort (including LS-IE, RS-IE, and CIED-IE), univariate analysis showed that embolism was associated with younger patient age, HIV infection, absence of cancer, presence of chronic liver disease, current intravenous drug abuse, RS-IE, larger vegetation size, *S. aureus* and *Enterococcus* species etiology (Table 3). The ROC curve analysis showed that a vegetation size ≥ 13 mm was significantly associated with the occurrence of emboli (Figure 2). Anticoagulant therapy at the onset of the IE episode was not associated with a significant difference in the frequency of embolic events; the same was true for antiaggregant therapy (Table 3). Multivariate analysis showed that the only independent predictors of embolism were prosthetic valve involvement, right-side localization, vegetation size (13 mm cut-off value) and *S. aureus* etiology (Table 4).

**Table 3:** Total cohort: univariate analysis of factors associated with embolism*

**Characteristics**	**IE episodes**	**IE episodes**	***p*** **≤ †**
	**with embolic**	**without embolic**	
	**events**	**events**	
Number of episodes of IE	499	957	
**Age (y)**	**62 (28 to 81)**	**67 (31 to 84)**	**0.0002**
Male	361 (72)	657 (69)	NS
Hemodialysis	10 (2)	19 (2)	NS
Diabetes mellitus	86 (17)	182 (19)	NS
**HIV infection**	**29 (6)**	**23 (2)**	**0.004**
**Cancer**	**46 (9)**	**129 (13)**	**0.03**
**Chronic liver disease**	**115 (23)**	**147 (15)**	**0.0005**
**Current intravenous drug abuse**	**74 (15)**	**57 (6)**	**0.0001**
Previous IE	38 (8)	80 (8)	NS
Chronic intravenous access	59 (12)	95 (10)	NS
Congenital heart disease	38 (8)	74 (8)	NS
Native valve predisposition	132 (26)	270 (28)	NS
Nosocomial IE	29 (6)	50 (5)	NS
Health care associated IE	107 (21)	227 (24)	NS
Community-acquired IE	363 (73)	680 (71)	NS
Native vs prosthetic valve	385 vs 114	732 vs 225	NS
Site of IE (n = 1282)			
Mitral vs aortic valve	166 vs 161	329 vs 359	NS
Bivalvular involvement (mitral and aortic)	39 (8)	78 (8)	NS
**Right-sided IE**	**60 (12)**	**29 (3)**	**0.0001**
CIED-IE	21 (4)	40 (4)	NS
Vegetations (n = 710)‡			
**Size of all vegetations (mm)**	**14 (6 to 30)**	**10 (4 to 26)**	**0.0001**
**≥ 10 mm**	**228 (79)**	**264 (63)**	**0.0001**
**≥ 15 mm**	**142 (49)**	**145 (35)**	**0.0001**
**≥ 20 mm**	**74 (26)**	**78 (19)**	**0.03**
Concomitant therapy at onset of IE			
Anticoagulants	102 (20)	228 (24)	NS
Antiaggregants	68 (14)	103 (11)	NS
Microbiology			
***Staphylococcus aureus***	**141 (28)**	**142 (15)**	**0.0001**
Viridans group streptococci	66 (13)	151 (16)	NS
Coagulase-negative staphylococci	52 (10)	95 (10)	NS
***Enterococcus *** **species**	**45 (9)**	**137 (1)**	**0.004**
*Streptococcus bovis*	44 (9)	98 (10)	NS
Polymicrobial	19 (4)	32 (3)	NS
Other microorganisms	16 (3)	45 (6)	NS
Other streptococci	16 (3)	43 (5)	NS
Fungi/yeasts	7 (1)	13 (1)	NS
HACEK	1 (0.2)	2 (0.2)	NS
Microbiology negative	92 (18)	199 (21)	NS

*Total cohort, N = 1456 episodes of infective endocarditis. Data reported as number, median (range, 5th to 95th percentile), or number (%). *Abbreviations:CIED-IE* infective endocarditis associated with a cardiac implantable electronic device, *HACEKHaemophilus* species, *Actinobacillus actinomycetemcomitans*, *Cardiobacterium hominis*, *Eikenella corrodens*, and *Kingella kingae*, *HIV* human immunodeficiency virus, *IE* infective endocarditis.

†NS, not significant (*p* > 0.05).

‡ IE episodes with vegetations of known size: 710 episodes (290 episodes with embolism and 420 episodes without embolism).

Bold numbers represent statistically significant differences.

**Table 4:** Total cohort: multivariate analysis of factors associated with embolism*

	**Odds ratio**	**95% CI**	**p ≤ †**
Age	1.0	1.0 – 1.0	NS
HIV infection	0.8	0.3 – 2.0	NS
Cancer	1.10	0.7 – 1.9	NS
Chronic liver disease	1.4	0.9 – 2.2	NS
Mitral vs aortic valve	1.0	0.7 – 1.3	NS
**Prosthetic vs native valve**	**1.8**	**1.2 – 2.9**	**0.011**
**Righ-sided IE**	**3.9**	**2.0 – 7.7**	**<0.0001**
**Size of vegetation ≥ 13 mm**	**1.9**	**1.3** – **2.6**	**<0.0001**
***Staphylococcus aureus***	**2.2**	**1.5** – **3.4**	**<0.0001**
*Coagulase-negative staphylococci*	1.6	1.0 – 2.6	NS
*Enterococcus spp*.	0.8	0.4 – 1.4	NS
Anticoagulant therapy	0.8	0.5 – 1.3	NS

*N = 1456 IE episodes. *Abbreviation:HIV* human immunodeficiency virus.

†NS, not significant (*p* > 0.05).

Bold numbers represent statistically significant differences.

In LS-IE, univariate analysis showed that embolism was associated with younger patient age, current intravenous drug abuse, larger vegetation size, *S. aureus* and *Enterococcus* species etiology (Additional file 3: Table S1). The ROC curve analysis showed that a vegetation size ≥ 13 mm was significantly associated with the occurrence of emboli (Additional file 4: Figure S1). Multivariate analysis showed that in LS-IE the only independent predictors of embolism were vegetation size (13 mm cut-off value; OR 2.1; 95% CI 1.5 – 2.8, p = 0.0001) and *S. aureus* etiology (OR 2.1; 95% CI 1.5 – 3.1, p = 0.0001) There were no significant differences in the frequency of embolism between mitral and aortic valves or between native and prosthetic valves. (Additional file 5: Table S2).

### Risk score

The two risk factors for LS-IE embolism that were identified with multivariate analysis (vegetation size ≥ 13 mm and *Staphylococcus aureus* etiology) were combined for designing the risk score. Because there was no marked difference in the odds ratios of the 2 prognostic variables, 1 point was assigned for the presence of each factor (total risk score range: minimum 0 points; maximum 2 points), this resulting in a three-tiered score:

•low risk category (no poor prognostic factor: etiology other than *S. aureus*, and vegetations size <13 mm);

•intermediate risk category (1 poor prognostic factor: *S. aureus* etiology or vegetations size ≥ 13 mm);

•high risk category (2 poor prognostic factors: *S. aureus* etiology and vegetations size ≥ 13 mm).

**Figure 3:**
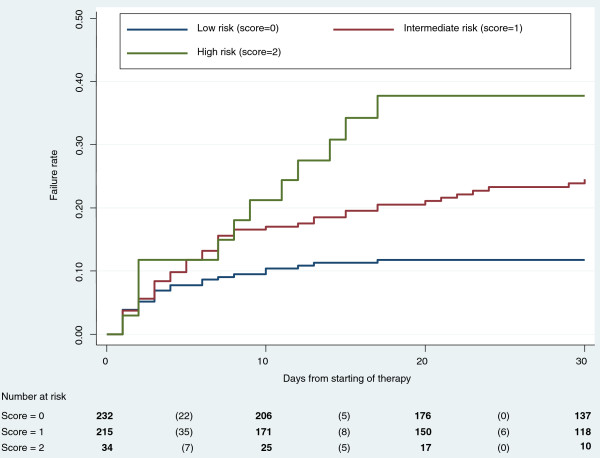
**Left-sided IE: Kaplan-Meier plots of failure rates (embolic events) for different risk categories.** Bold numbers: number of patients at risk at any given time. Numbers in parentheses: number of embolic events that occurred in each time interval.

•The 30-day cumulative incidence of embolism varied significantly across the different risk categories: 11.8% (95% CI 7.2 – 19.2) in the low risk category, 24.5% (95% CI 20.3 – 37.0) in the intermediate risk category, 37.7 (95% CI 22.1 – 64.9) in the high risk category. Kaplan-Meier plots showed that the 3 risk categories differed significantly from each other by log-rank test and test for trend (p ≤ 0.001) (Figure 3, Table 5).

**Table 5:** Left-sided IE: 30-day probability (%) of embolic events per risk category

**Time from start of therapy (d)**	**Low (0)**	***Intermediate *** **(1)**	***High *** **(2)**
0	11.78	24.54	37.73
1	7.90	20.82	34.79
2	6.61	18.95	25.97
3	4.88	16.13	25.97
4	4.01	14.70	25.97
5	4.01	12.78	25.97
6	3.14	11.34	25.97
7	2.71	8.95	22.81
15	0.48	5.02	3.46
30	0.00	0.00	0.00

Note: numbers represent the probability (%) of embolism at the end of a given time interval. Time 0 values include embolic events occurred on the first calendar day of therapy; day 1 values represent the risk at the end of the first calendar day of therapy.

## Discussion

Embolism is a common complication of infective endocarditis. The cumulative incidence of clinically overt embolic events in our series (34.3%) is within the range observed in most published series, notwithstanding differences in definitions and enrollment criteria between studies [6,7,12,13,22,26,29,30]. More in detail, the incidence of embolization observed in our study in LS-NVE (31.4%), LS-PVE (33.6%), RS-IE (67%), CIED-IE (34.4%) is similar to what has been reported from most contemporary studies: a high incidence of embolic events in RS-IE, and an incidence in the range 20-33% for CIED-associated IE; it has to be noted that in our study, in contrast with other published series, we did not observe significant differences in the frequency of embolic events between mitral and aortic valve localization, and between LS-NVE and LS-PVE [8,15].

It is well known that embolic episodes occur early in the course of the disease, often before the diagnosis of IE is established and antimicrobial therapy is started; our findings confirm this, with a large part of the embolic events (39.6%) occurring in the days preceding the diagnosis or on the day of diagnosis [3,8,15]. The present study confirms that the risk of embolism decreases markedly after the first week of antimicrobial therapy; this seems to support previous recommendations against stroke prevention as the only indication for valvular surgery after 1 week of therapy [15].

An embolic event may predict the risk of further embolic events and neurologic complications, and this may have an effect on decision making [3,14]. In our series, central nervous system embolism was preceded by symptomatic peripheral embolism in just 25 patients, this apparently limiting the utility of peripheral embolism as a predictor for CNS embolic complications. These 25 patients might point to a missed opportunity for surgical prevention of embolism, but it has to be noted that in the SEI study data regarding the possible presence of major contraindications for surgery were not systematically collected.

Many papers have reported on the characteristics of vegetations which are predictive of the risk of embolism [1,3,7,12,25,26,31]; lacking a standardized echocardiographic study of our patients, the analysis was restricted to the size of the vegetations, which is a major determinant of the embolic risk, as underlined by two meta-analyses [32,33]. The cut-off value of 13 mm derived by our cumulative analysis (and confirmed by an analysis restricted to the LS-IE episodes) is within the range commonly discussed in the literature (10 mm or 15 mm for LS-IE, and 20 mm for RS-IE) [3,7,12,25,26,31].

*S. aureus* has been associated with an increased risk of embolic events; our data clearly confirm this, with an OR for *S. aureus* versus all other microbial agents of 2.2 [3,7,8,15,34]. A few papers have also reported on a higher incidence of embolic complications in *Streptococcus bovis* IE: our analysis of 142 *S. bovis* IE episodes did not confirm these findings; it has to be noted that in the report by Pergola et al. (40 *S. bovis* episodes), a significant difference was observed when comparing *S. bovis* with other streptococci, but not when comparing *S. bovis* with other pathogens; and Tripodi et al. found a significant difference when comparing *S. bovis* IE (30 episodes) with IE due to other etiological agents (p = 0.002 at univariate analysis); however, patients with *S. bovis* IE were older and had a higher rate of bivalvular involvement [7,35,36]. High rates of embolic events have been described in fungal endocarditis (43% in 21 cases of PVE-IE reported by Boland); in our series (20 fungal episodes) we did not observe a significant difference when comparing fungal IE episodes with episodes due to other pathogens [37-39].

In our series age, sex and a number of predisposing and comorbidity conditions were not found to be associated with the occurrence of embolic events, and were not included in the score; this is in contrast with what has recently been proposed by Hubert et al., which included in their risk calculator not only vegetation size (10 mm cut-off) and *S. aureus* etiology, but also age, diabetes, previous embolism and atrial fibrillation [3].

Despite the large sample size in the present study, only 2 factors were significantly associated with the occurrence of embolic events in LS-IE: vegetation size ≥ 13 mm and *S. aureus* etiology. These variables were incorporated into a 3-tiered risk score; the 3 categories of the scoring system were associated with clinically relevant differences in the risk of developing embolic events. Combining the risk category with the number of days on antimicrobials could be used to assess the probability of embolism (as shown in Table 5), in order to compare the embolic risk with the risks associated with surgery, making it possible to individualize a patient’s evaluation, especially during the crucial first days of the clinical course. However, differences in patient populations, clinical settings, and models of care may affect the predictive value of the score. Therefore, the clinical use of the scoring system will be possible only following successful external validation.

Our study has inherent limitations: firstly, the participant centers were secondary and tertiary care units, where a major interest in the field of IE had developed; yet, patients with IE are admitted all over the country to Infectious Diseases, Internal Medicine, Cardiology, Cardiac Surgery wards and to intensive care units, and a referral bias has to be taken in account; in fact, our data do not represent the wide range of clinical conditions and patterns of treatment which are observed across Italy, and there is clearly the need for large, population-based studies, which could better describe the full spectrum of the disease, as currently observed and treated in our country. Secondly, echocardiographic evaluation was left to local expertise, without a centralized standardization or supervision: this may have limited our ability to identify characteristics of the vegetations that may be associated with the occurrence of embolic events. Finally, embolization was diagnosed at the different institutions in accordance with current clinical practice, and therefore differences in the diagnostic approach may have reduced the discriminatory power of our analysis.

## Conclusions

Embolism occurs early in the course of IE; the steep decline of the risk of embolization immediately after antibiotics initiation allows only a narrow time window for the surgical prophylaxis of embolic complications. In this challenging clinical context, the simple score system described in the present study, if externally validated, might contribute to a timely and individualized assessment of the embolic risk.

## Competing interests

No financial support was obtained for this work.

The authors declare that they have no competing interests.

## Authors’ contributions

MR designed the study; VR managed the database; AC performed all statistical analyses; MR prepared the initial draft of the manuscript; MBP, EDM, and AP, revised the manuscript and made important content contributions; all authors were involved in manuscript review and editing; all authors, with the exception of AC, contributed to pts’ enrolment, and to the implementation of the SEI study. All authors read and approved the final manuscript.

## Pre-publication history

The pre-publication history for this paper can be accessed here:

http://www.biomedcentral.com/1471-2334/14/230/prepub

## Supplementary Material

Additional file 1 Embolic events and in-hospital mortality. Click here for file

Additional file 2 Embolic events and surgery. Click here for file

Additional file 3: Table S1 Left-sided IE: univariate analysis of factors associated with embolism. Click here for file

Additional file 4: Figure S1 Left-sided IE: size of vegetation. Receiver operating characteristic analysis for the prediction of embolic events, based on vegetation size. Click here for file

Additional file 5: Table S2 Left-sided IE: multivariate analysis of factors associated with embolism. Click here for file
