# Supplementary material for: Predicting the occurrence of embolic events: an analysis of 1456 episodes of infective endocarditis from the Italian Study on Endocarditis (SEI)
Source: BMC Infect Dis. 2014 Apr 29;14:230. doi: 10.1186/1471-2334-14-230 (PMC4101861; doi:10.1186/1471-2334-14-230)
Supplement: Additional file 1 — Embolic events and in-hospital mortality. [file 1471-2334-14-230-S1.docx]

**ADDITIONAL MATERIAL**

**Appendix 1. Embolic events and in-hospital mortality**

The overall in-hospital mortality was 201/1456 (13.8%), as follows: LS-NVE 119/967 (12.3%), LS-PVE 67/339 (19.8%), RS-IE 8/89 (9.0%), CIED-IE 7/61 (13.1%). In IE episodes not complicated by embolic events, in-hospital death was 107/957 (11.2%), versus 94/499 (18.8%) in embolic episodes. The mortality rate was 43/176 (24.4%) in episodes with ischemic stroke, 15/31 (48.4%) in episodes with hemorrhagic stroke, 33/269 (12.3%) in episodes complicated by peripheral embolism. The association of embolization with in-hospital death was stronger for PVE: non-embolic PVE mortality was 31/225 (13.8%), versus 36/114 (31.6%)
