# Supplementary material for: Predicting the occurrence of embolic events: an analysis of 1456 episodes of infective endocarditis from the Italian Study on Endocarditis (SEI)
Source: BMC Infect Dis. 2014 Apr 29;14:230. doi: 10.1186/1471-2334-14-230 (PMC4101861; doi:10.1186/1471-2334-14-230)
Supplement: Additional file 2 — Embolic events and surgery. [file 1471-2334-14-230-S2.docx]

**ADDITIONAL MATERIAL**

**Appendix 2. Embolic events and surgery**

Cardiac surgery was performed during the actual IE episode in 593 IE cases (40.7%), with a median time from diagnosis to surgery of 19 days (interquartile range, 9 to 46 days); the occurrence of an embolic event was not associated with a higher rate of surgical procedures: there were 202/499 (40.5%) surgical procedures in patients with embolism versus 391/957 (40.8%) procedures in patients without embolism. The prevention of embolism was reported as an indication for surgery in 114 episodes (in 38 IE episodes in order to prevent a possible first embolic event, in 76 IE episodes following one or more embolic events). For the 202 IE episodes with embolism which had surgery, surgery was performed before any embolic event in 31 instances, following peripheral embolism in 89 instances, following CNS embolism in 82 cases (9 TIAs: embolism to surgery median time 33 days; 65 ischemic strokes: embolism to surgery median time 16 days; 8 hemorrhagic strokes: embolism to surgery median time 32 days).
