# Supplementary material for: Predicting the occurrence of embolic events: an analysis of 1456 episodes of infective endocarditis from the Italian Study on Endocarditis (SEI)
Source: BMC Infect Dis. 2014 Apr 29;14:230. doi: 10.1186/1471-2334-14-230 (PMC4101861; doi:10.1186/1471-2334-14-230)
Supplement: Additional file 3: Table S1 — Left-sided IE: univariate analysis of factors associated with embolism. [file 1471-2334-14-230-S3.docx]

**Supplementary table 1. Left-sided IE: univariate analysis of factors associated with embolism***

| **Characteristics** | **IE Episodes With**  **Embolic Events** | **IE Episodes Without**  **Embolic Events** | ***p*  ≤ †** |
| --- | --- | --- | --- |
| Number of episodes of IE | 418 | 888 |  |
| **Age (y)** | **64 (28 to 81)** | **67 (33 to 83)** | **0.0002** |
| Male | 301 (72) | 608 (69) | NS |
| Hemodialysis | 8 (2) | 17 (2) | NS |
| Diabetes mellitus | 73 (17) | 170 (19) | NS |
| HIV infection | 19 (5) | 21 (2) | NS |
| Cancer | 40 (10) | 119 (13) | NS |
| Chronic liver disease | 80 (19) | 138 (16) | NS |
| **Current intravenous drug abuse** | **43 (10)** | **52 (6)** | **0.01** |
| Previous IE | 32 (8) | 75 (8) | NS |
| Chronic intravenous access | 46 (11) | 90 (10) | NS |
| Congenital heart disease | 33 (8) | 68 (8) | NS |
| Native valve predisposition | 132 (32) | 270 (30) | NS |
| Nosocomial IE | 24 (6) | 46 (5) | NS |
| Health care associated IE | 95 (23) | 205 (23) | NS |
| Community-acquired IE | 299 (72) | 637 (72) | NS |
| Native vs prosthetic valve | 385 vs 114 | 732 vs 225 | NS |
| Vegetations (n = 607)* |  | | |
| **Size of all vegetations (mm)** | **14 (5 to 27)** | **10 (4 to 25)** | **0.0001** |
| **≥ 10 mm** | **173 (76)** | **233 (61)** | **0.0002** |
| **≥ 15 mm** | **106 (47)** | **121 (32)** | **0.0003** |
| ≥ 20 mm | 48 (21) | 61 (16) | NS |
| Concomitant therapy at onset of IE |  | | |
| Anticoagulants | 98 (24) | 222 (26) | NS |
| Antiaggregants | 59 (14) | 99 (11) | NS |
| Microbiology |  | | |
| ***Staphylococcus aureus*** | **109 (26)** | **125 (14)** | **0.0001** |
| Viridans group streptococci | 58 (14) | 143 (16) | NS |
| Coagulase-negative staphylococci | 45 (11) | 84 (10) | NS |
| ***Enterococcus* species** | **41 (10)** | **129 (15)** | **0.02** |
| *Streptococcus bovis* | 39 (9) | 95 (11) | NS |
| Other streptococci | 15 (4) | 38 (4) | NS |
| Polymicrobial | 14 (3) | 30 (3) | NS |
| Other microorganisms | 13 (3) | 43 (5) | NS |
| Fungi | 7 (2) | 13 (2) | NS |
| HACEK | 0 (0) | 2 (0.2) | NS |
| Microbiology negative | 77 (18) | 186 (21) | NS |

* N = 1306 episodes of left-sided IE. Data reported as number, median (range, 5th to 95th percentile), or number (%). Abbreviations: HACEK, *Haemophilus* species, *Actinobacillus actinomycetemcomitans*, *Cardiobacterium homini*s, *Eikenella* *corrodens*, and *Kingella kingae*; HIV, human immunodeficiency virus; IE, infective endocarditis.

† NS, not significant (*p* > 0.05)

‡ IE episodes with vegetations of known size: 607 episodes (227 episodes with embolism and 380 episodes without embolism).

**Supplementary table 2. Left-sided IE: multivariate analysis of factors associated with embolism***

| **Variable** | **Hazard Ratio** | **95% Confidence Interval** | ***p*  ≤ †** |
| --- | --- | --- | --- |
| Age | 1.0 | 1.0 – 1.0 | NS |
| Mitral vs aortic valve | 0.8 | 0.6 – 1.1 | NS |
| Prosthetic vs native valve | 1.5 | 1.0 – 2.2 | NS |
| **Size of vegetation ≥ 13 mm** | **2.1** | **1.5 – 2.8** | **0.0001** |
| ***Staphylococcus aureus*** | **2.1** | **1.5 – 3.1** | **0.0001** |
| *Enterococcus* species | 0.8 | 0.5 **–** 1.3 | NS |
| Anticoagulant therapy | 1.5 | 0.9 **–** 2.3 | NS |

* N = 1306 episodes of left-sided IE. Abbreviations: HIV, human immunodeficiency virus.

† NS, not significant (*p*  > 0.05)
