# Supplementary figures and images for: Predicting the occurrence of embolic events: an analysis of 1456 episodes of infective endocarditis from the Italian Study on Endocarditis (SEI)
Source: BMC Infect Dis. 2014 Apr 29;14:230. doi: 10.1186/1471-2334-14-230 (PMC4101861; doi:10.1186/1471-2334-14-230)

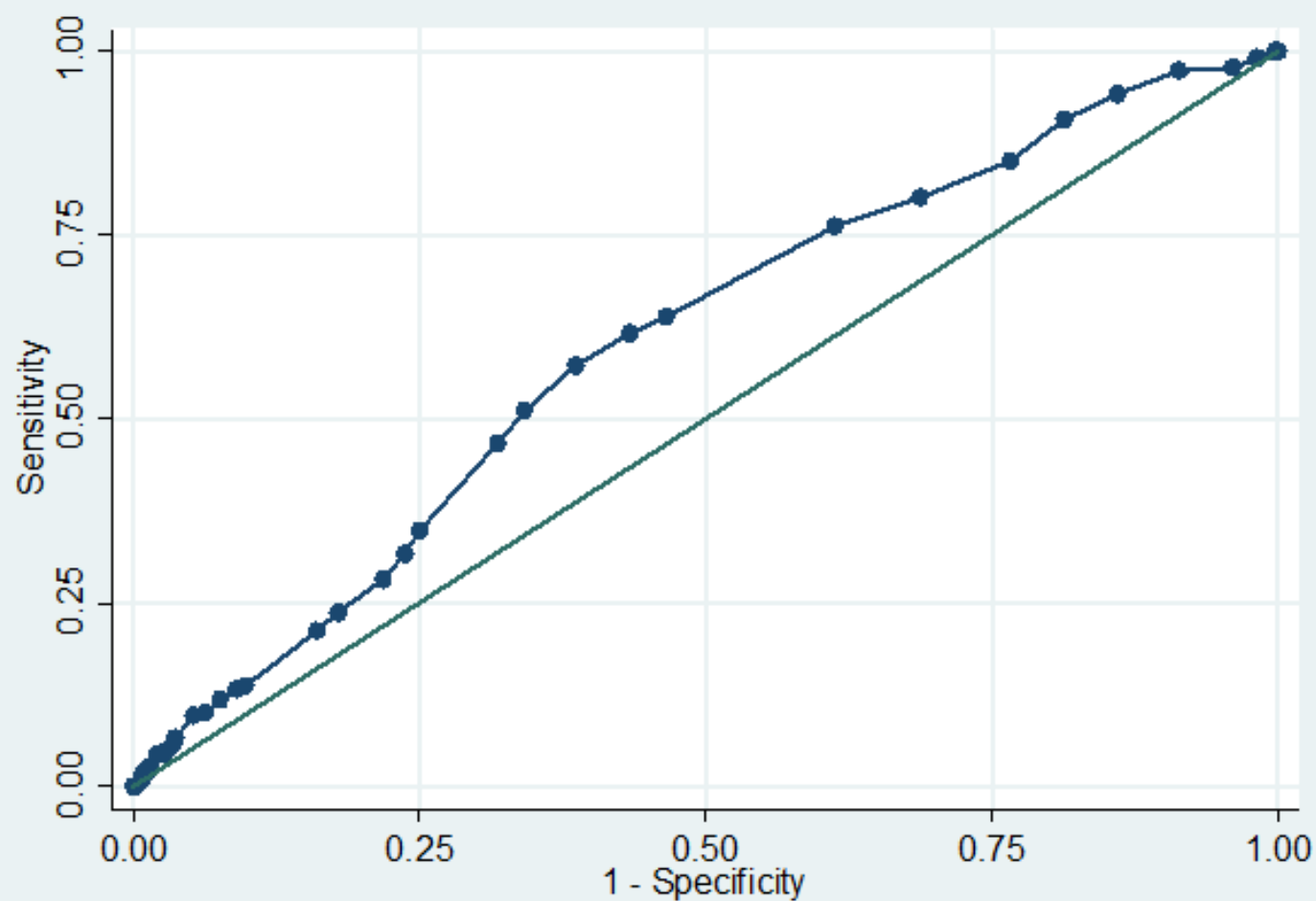

Area under ROC curve = 0.6019

Supplement: Additional file 4: Figure S1 — Left-sided IE: size of vegetation. Receiver operating characteristic analysis for the prediction of embolic events, based on vegetation size. [file 1471-2334-14-230-S4.pdf]
