# Supplementary material for: Predicting the occurrence of embolic events: an analysis of 1456 episodes of infective endocarditis from the Italian Study on Endocarditis (SEI)
Source: BMC Infect Dis. 2014 Apr 29;14:230. doi: 10.1186/1471-2334-14-230 (PMC4101861; doi:10.1186/1471-2334-14-230)
Supplement: Additional file 5: Table S2 — Left-sided IE: multivariate analysis of factors associated with embolism. [file 1471-2334-14-230-S5.docx]

**Supplementary table 2. Left-sided IE: multivariate analysis of factors associated with embolism***

| **Variable** | **Odds ratio** | **95% Confidence Interval** | ***p*  ≤ †** |
| --- | --- | --- | --- |
| Age | 1.0 | 1.0 – 1.0 | NS |
| Mitral vs aortic valve | 0.8 | 0.6 – 1.1 | NS |
| Prosthetic vs native valve | 1.5 | 1.0 – 2.2 | NS |
| **Size of vegetation ≥ 13 mm** | **2.1** | **1.5 – 2.8** | **0.0001** |
| ***Staphylococcus aureus*** | **2.1** | **1.5 – 3.1** | **0.0001** |
| *Enterococcus* species | 0.8 | 0.5 **–** 1.3 | NS |
| Anticoagulant therapy | 1.5 | 0.9 **–** 2.3 | NS |

* N = 1306 episodes of left-sided IE. Abbreviations: HIV, human immunodeficiency virus.

† NS, not significant (*p*  > 0.05)
